# Predictors of Detectable Viremia, Outcomes, and Implications for Management of People Living With HIV Who Are Receiving Antiretroviral Therapy in Southern Nigeria

**DOI:** 10.1093/ofid/ofad562

**Published:** 2023-11-09

**Authors:** Uduak U Akpan, Esther N Nwanja, Titilope Badru, Otoyo E Toyo, Augustine M Idemudia, Olusola Sanwo, Pius Nwaokoro Okeke, Bala Gana, Saade Idem, Helen M Idiong, Hadiza G Khamofu, Moses H Bateganya

**Affiliations:** Achieving Health Nigeria Initiative, Akwa Ibom, Nigeria; Achieving Health Nigeria Initiative, Akwa Ibom, Nigeria; FHI 360, Abuja, Nigeria; Achieving Health Nigeria Initiative, Akwa Ibom, Nigeria; Achieving Health Nigeria Initiative, Akwa Ibom, Nigeria; FHI 360, Abuja, Nigeria; FHI 360, Abuja, Nigeria; Achieving Health Nigeria Initiative, Akwa Ibom, Nigeria; Achieving Health Nigeria Initiative, Akwa Ibom, Nigeria; Achieving Health Nigeria Initiative, Akwa Ibom, Nigeria; FHI 360, Abuja, Nigeria; FHI 360, Abuja, Nigeria; FHI 360, Durham, North Carolina, USA

**Keywords:** detectable viremia, Nigeria, people living with HIV, undetectable viral load, virologic failure

## Abstract

**Background:**

This study examined the prevalence and factors associated with detectable viremia, as well as clinical outcomes among people with HIV (PWH) receiving antiretroviral therapy (ART) who initially achieved viral suppression in 2 southern states in Nigeria.

**Methods:**

The retrospective cohort study used data from the electronic medical records of 96 comprehensive ART centers. PWH were followed up who achieved viral suppression (viral load [VL] ≤50 copies/mL) upon starting ART based on the first VL test. We examined the presence of detectable viremia in follow-up VL results, graded by the absolute VL count from the second and third consecutive VL tests as follows: transient viremia (second follow-up VL, 51–999 copies/mL; third, ≤50 copies/mL), persistent viremia (second follow-up VL, 51–999 copies/mL or ≥1000 copies/mL; third, >50 copies/mL), and virologic failure (second and third follow-up VL, >1000 copies/mL). We analyzed demographic and clinical factors associated with detectable viremia using logistic regression analysis on Stata 14.

**Results:**

Overall, 15 050 PWH had achieved viral suppression following ART initiation (median age, 34 years; 71.3% female). On follow-up, 3101 (20.6%) had a viremic event: 11.6%, transient viremia; 8.8%, persistent viremia; 0.2%, virologic failure. Shorter duration of ART (*P* < .001), being 0 to 14 years of age (*P* < .001), and not being enrolled in a differentiated service delivery model (*P* < .001) were significantly associated with detectable viremia.

**Conclusions:**

Our study shows that people who initially attain vial suppression upon starting ART remain at risk of detectable viremia.

## Teaser

This article provides further evidence for the need for enhanced monitoring and management of people living with HIV even after they have achieved viral suppression. It highlights priority subgroups for enhanced care, including those not enrolled in a differentiated service delivery model, those aged 0 to 14 years, and people living with HIV who have been undergoing antiretroviral therapy for a shorter period.

The main goal for antiretroviral therapy (ART) is to achieve viral suppression at the individual and population levels [1]. Globally, 27.5 million people with HIV (PWH) were undergoing ART, and approximately two-thirds were estimated to be virally suppressed as of 2020 [2]. Sub-Saharan Africa is the most affected region, accounting for two-thirds of all new HIV infections worldwide [2]. Nigeria has the second-largest HIV epidemic in the world, with an estimated 1.9 million PWH as of 2020 [3]. By 2021, 90% of PWH were aware of their HIV status; of those, 86% were receiving ART; and among those recipients, 72% were virally suppressed [3].

Sustained ART maintains maximal suppression of the viral load (VL; ie, undetectable VL), restores or preserves immunologic function, improves quality of life, and reduces HIV-related morbidity and mortality [4, 5]. Maintaining an undetectable VL level is one of the important ways of preventing the development of drug resistance and ongoing HIV transmission [6–10].

PWH who achieve undetectable VL levels may subsequently experience transient or persistent viremia [11–14]. Persistent viremia has been associated with increased vulnerability to opportunistic infections, treatment failure, drug resistance, and HIV transmission [15, 16]. Data from high-income countries show a need for ongoing VL monitoring even after achieving viral suppression. Laprise et al studied the long-term effect of persistent low-level viremia among a cohort of PWH, while Gunn et al showed that a third of participants experienced virologic failure despite attaining sustained viral suppression [17, 18]. However, there are limited data on the outcome of clients who attain undetectable VL levels in low- and middle-income countries.

This study describes the prevalence of detectable viremia among people who initially achieve viral suppression. We also describe demographic and clinical factors associated with detectable viremia as well as their clinical outcomes.

## METHODS

### Study Design and Population

We conducted a secondary analysis of program data for PWH receiving ART from 96 health facilities in Akwa Ibom and Cross River States in Nigeria. We analyzed those who had an undetectable VL (≤50 copies/mL) as of December 2019 and 2 follow-up VL results (at least 6 months apart in line with the national guideline on VL monitoring) [1].

### Study Setting

The study was conducted in 2 states in Nigeria (Akwa Ibom and Cross River States), where the United States Agency for International Development funded the EpiC project (Meeting Targets and Maintaining Epidemic Control) to provide comprehensive HIV care and treatment services in 154 health facilities (2 tertiary, 48 secondary, and 104 primary health facilities) and 101 community pharmacies. The project deployed 67 community ART management teams [19] (“Composition of the Community ART Management Teams” section), which comprised clinicians, pharmacists, laboratory scientists, counselor testers, case managers, and community mobilizers who provided HIV care and treatment services at the community level in line with the national task-shifting policy (20).

ART services, including treatment monitoring, was provided according to the “National Guideline for HIV Prevention, Treatment and Care” [1]. Clients who were eligible for VL had their blood sample collected at the service delivery points, processed to separate the plasma, and transported to a central laboratory at the University of Uyo Teaching Hospital. Here a polymerase chain reaction machine analyzed the plasma samples, and results were sent back to the originating health facility through an online Laboratory Information Management System. The platforms used for VL sample analysis were the Roche COBAS AmpliPrep (daily capacity of 210 samples), Abbott (daily capacity of 465 samples), and Hologic Panther (daily capacity of 658 samples), and each had a low detection limit (<40 copies/mL).

### Data Collection

Data for this study were obtained from the electronic medical records of the Lafiya Management Information System in each health facility [21]. Abstracted data included age, sex, education, marital status, occupation, date and setting of ART initiation, World Health Organization clinical staging, differentiated service delivery (DSD) model [22], and ART regimen.

ART regimen (first line only) was classified into the following categories: HIV integrase inhibitors (tenofovir/lamivudine/dolutegravir and abacavir/lamivudine/dolutegravir), protease inhibitors (abacavir/lamivudine/lopinavir/ritonavir and tenofovir/lamivudine/lopinavir/ritonavir), and nonnucleoside reverse transcriptase inhibitors (tenofovir/lamivudine/efavirenz and tenofovir/lamivudine/nevirapine).

The date of the initial VL test following ART initiation was taken as the baseline. We categorized virologic outcomes after the first VL test (first follow-up test) based on the results of the 2 follow-up VL tests:

Maintained durable viral suppression: the 2 follow-up VL results were ≤50 copies/mL.

Transient viremia: the second follow-up VL was 51 to 999 copies/mL, but the third result was ≤50 copies/mL.

Persistent viremia: the second follow-up VL was either 51 to 999 or ≥1000 copies/mL, but the third result was >50 copies/mL.

Virologic failure: the second and third follow-up VL results were >1000 copies/mL (Table 1).

PWH reviewed were classified at follow-up as such: active (if the client maintained treatment until the end of the review period in March 2023), deceased, stopped treatment, or lost to follow-up (if the client had not picked up an ART refill for ≥28 days from the last expected refill appointment within the review period).

**Table 1.** Categorization of VL Event Based on Absolute VL Count

	VL Result, Copies/mL		
	First	Second	Third
Durable viral suppression	Undetectable, <50	Undetectable, <50	Undetectable, <50
Transient viremia	Undetectable, <50	Unsuppressed VL, ≥1000	Undetectable, <50
	Undetectable, <50	Detectable VL, 50–999	Undetectable, <50
Persistent viremia	Undetectable, <50	Detectable VL, 50–999	Detectable, ≥50
	Undetectable, <50	Unsuppressed, ≥1000	Detectable, ≥50
Virologic failure	Undetectable, <50	Unsuppressed, ≥1000	Unsuppressed, ≥1000

Abbreviation: VL, viral load.

### Data Analysis

Descriptive statistics were used to analyze categorical variables and summarized by frequency, while continuous skewed variables were summarized as median and IQR. VL events (durable/sustained suppression, transient viremia, persistent viremia, and virologic failure) were summarized descriptively to determine distribution by the various client demographic and clinical characteristics. We used logistic regression analysis to calculate unadjusted and multivariable-adjusted odds ratios and 95% CIs to determine factors associated with viremia. All *P* values <.05 were statistically significant.

Chi-square statistics was then used to compare treatment outcome by ranges of VL. We dichotomized treatment outcome into “active” vs “treatment interruption.” All statistical analyses were performed in Stata 15 (StataCorp LLC).

### Ethical Approval

Permission to conduct a secondary analysis on these routine data was provided by Family Health International's Office of International Research Ethics (approval 1851459-1). The ethics committee waived the requirement for informed consent by participants, as VL testing is part of the Ministry of Health’s routine standard of care for monitoring HIV treatment response. Also, data from the electronic medical records used for analysis in the study were fully anonymized, as clients’ names were excluded in the abstraction of data.

## RESULTS

**Figure 1.**
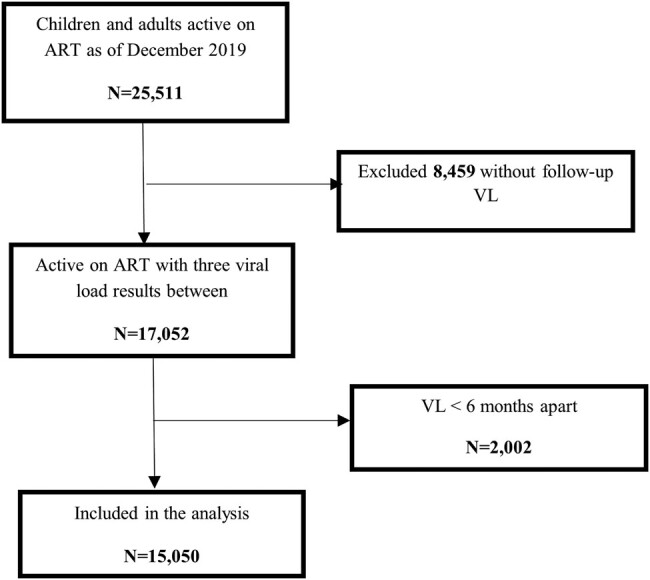
Study process of cohort identification for children and adults living with HIV with detectable viremia. ART, antiretroviral therapy; VL, viral load.

Of the 25 511 records of children and adults active on ART as of December 2019, 17 052 records had at least 3 VL results within the period of review. An overall 2002 records were excluded because they contained VL results <6 months apart (VL after enhanced adherence counseling after a VL ≥1000 copies/mL). A total of 15 050 (64%) records categorized as unsuppressed were included in this analysis, (Figure 1).

The sociodemographic and baseline clinical characteristics of the 15 050 clients are shown in Table 2. The median age was 34 years (IQR, 28–42); 71.3% of clients were female; 64.6% had secondary education; 60.8% were married; and 59.8% were unemployed. Furthermore, 36.8% were asymptomatic at ART initiation; 90.6% were receiving ART through one of several DSD models; and 96.3% had been diagnosed and initiated ART in a community setting.

**Table 2.** Sociodemographic and Clinical Characteristics of Adults and Children Living With HIV Who Were Virally Suppressed in Southern Nigeria (N = 15 050)

Variable	No. (%)
Sex (N = 15 050)	
Male	4322 (28.7)
Female	10 728 (71.3)
Age, y	
0–14	451 (3.0)
15–24	1749 (11.6)
25–64	12 692 (84.3)
≥65	158 (1.1)
Age, y, median (IQR)	34 (28–42)
Education^a^ (n = 12 254)	
None	1045 (8.5)
Primary	3289 (26.8)
Secondary	7922 (64.6)
Marital status^a^ (n = 14 216)	
Never married	4190 (29.5)
Married	8644 (60.8)
Previously married	1382 (9.7)
Occupation^a^ (n = 13 082)	
Employed	4324 (33.1)
Unemployed	7822 (59.8)
Student	936 (7.2)
WHO clinical stage^a^ (n = 14 800)	
I	5440 (36.8)
II	4342 (29.3)
III	4758 (32.2)
IV	260 (1.8)
Regimen^b^ (n = 14 695)	
HIV integrase inhibitors	14 656 (99.7)
Protease inhibitors	32 (0.2)
NNRTIs	7 (0.1)
Time undergoing ART, y	
1.5–3	3487 (23.2)
4–5	4666 (31.0)
6–10	4593 (30.5)
>10	2304 (15.3)
Enrollment in DSD	
Yes	13 636 (90.6)
No	1414 (9.4)
ART initiation setting^a^ (n = 14 976)	
Facility	562 (3.8)
Community	14 414 (96.3)

Abbreviations: ART, antiretroviral therapy; DSD, differentiated service delivery; NNRTI, nonnucleoside reverse transcriptase inhibitor; WHO, World Health Organization.

^a^With missing data.

^b^Analyzed for first-line ART only.

### Virologic Outcomes

**Figure 2.**
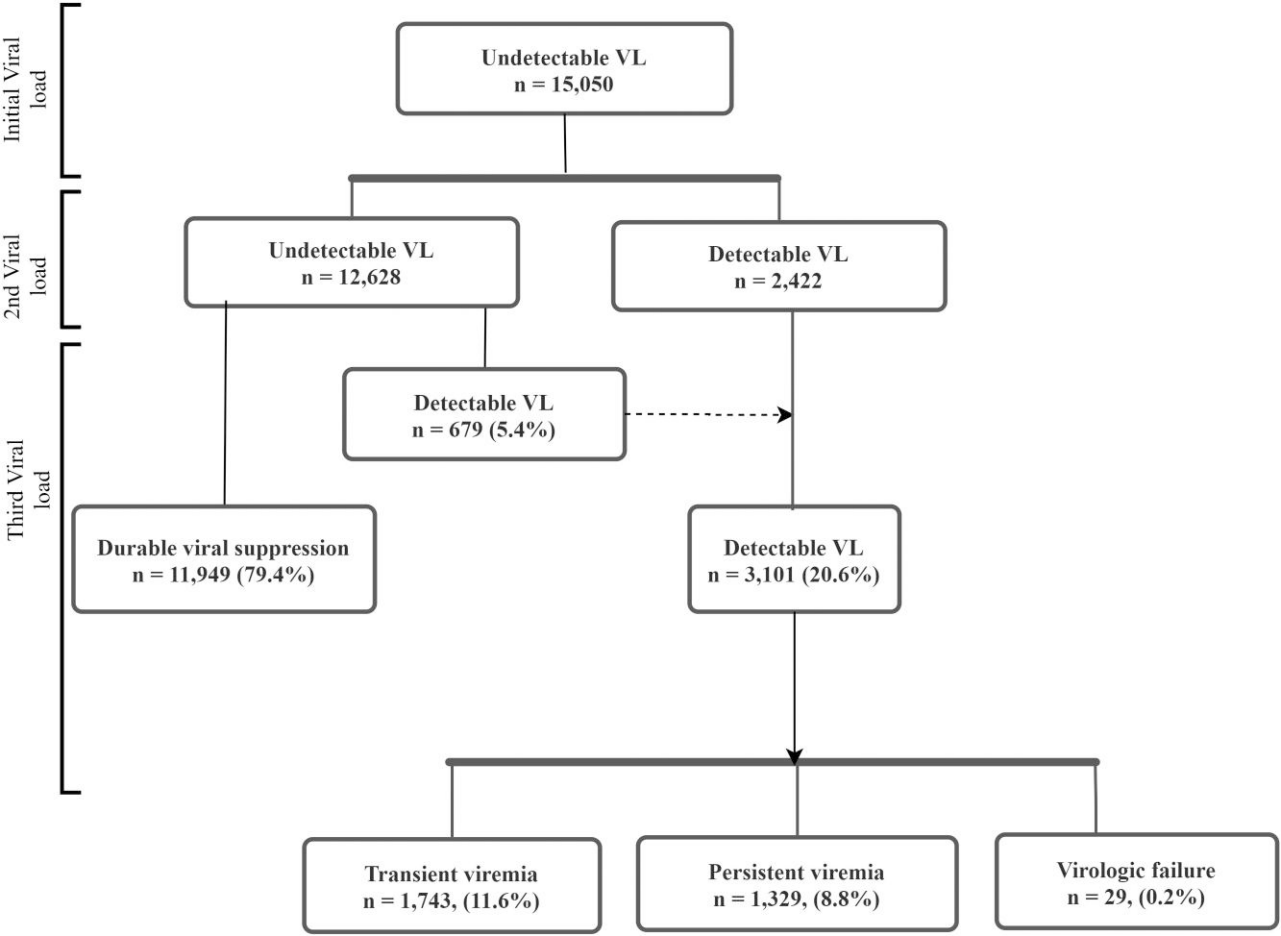
Patterns of detectable viral load (VL): follow-up measurements after initial undetectable viral load levels.

Figure 2 describes clients who were virally suppressed and those who developed viremic events during follow-up. Overall, 79.4% of clients with an undetectable VL at initial viral measurement remained virally suppressed, while 20.6% had detectable viremia. Among those with detectable viremia, 11.6% had transient viremia, while 8.8% and 0.2% experienced persistent viremia and virologic failure, respectively.

Table 3 shows VL outcomes by client characteristics. There was significant variation in the distribution of viremic events across the age bands, time undergoing ART, and enrollment in DSD. Up to 71.4% of PWH aged 0 to 14 years maintained durable viral suppression, as opposed to 79.4%, 79.7%, and 79.9% among those aged 15 to 24, 25 to 64, and ≥65 years, respectively. Greater than 80% of PWH undergoing ART for >10 years and 6 to 10 years maintained viral suppression, as compared with 79.6% and 76.0% among those receiving ART for 4 to 5 years and 1.5 to 3 years. Notably, 81.4% of PWH enrolled in a DSD model achieved or maintained a viral suppression outcome, as opposed to 59.7% among those not involved in a DSD model.

**Table 3.** Viral Load Outcomes by Client Characteristics for Clients With Initial Undetectable Viral Load in Southern Nigeria (N = 15 050)

		Detectable Viremia				
	Durable Viral Suppression	Transient Viremia	Persistent Viremia	Virologic Failure	χ^2^	*P* Value
Sex					1.2	.75
Male	3429 (79.4)	502 (11.6)	380 (8.8)	11 (0.2)		
Female	8520 (79.3)	1241 (11.6)	949 (8.8)	18 (0.3)		
Age, y					22.5	.007
0–14	322 (71.4)	77 (17.1)	50 (11.1)	2 (0.4)		
15–24	1388 (79.4)	199 (11.4)	158 (9.0)	4 (0.2)		
25–64	10 119 (79.7)	1447 (11.4)	1103 (8.7)	23 (0.2)		
≥65	120 (79.9)	20 (12.7)	18 (11.4)	0 (0)		
Education					14.9	.021
None	816 (78.1)	126 (12.1)	102 (9.8)	1 (0.1)		
Primary	2561 (77.9)	433 (13.2)	293 (8.9)	2 (0.1)		
Secondary	6329 (79.9)	877 (11.2)	687 (8.7)	19 (0.3)		
Marital status					7.3	.29
Never married	3295 (78.6)	488 (11.6)	399 (9.5)	8 (0.2)		
Married	6880 (79.6)	153 (11.1)	128 (9.3)	0 (0)		
Previously married	1101 (79.7)	1007 (11.6)	737 (8.5)	20 (0.2)		
Occupation					8.6	.19
Employed	3471 (80.3)	469 (10.8)	374 (8.6)	10 (0.2)		
Unemployed	6145 (78.6)	967 (12.4)	697 (8.9)	13 (0.2)		
Student	754 (80.6)	102 (10.9)	79 (8.4)	1 (0.1)		
WHO stage					7.5	.59
I	4336 (79.7)	608 (11.2)	487 (9.0)	9 (0.2)		
II	3432 (79.0)	496 (11.4)	405 (9.3)	9 (0.2)		
III	3774 (79.3)	581 (12.2)	394 (8.3)	9 (0.2)		
IV	204 (78.5)	35 (13.5)	20 (7.7)	1 (0.4)		
Regimen					5.1	.53
HIV integrase inhibitors	11 677 (79.7)	1688 (11.5)	1263 (8.6)	28 (0.2)		
Protease inhibitors	24 (75.0)	2 (6.3)	6 (18.8)	0 (0)		
NNRTIs	5 (71.4)	1 (14.3)	1 (14.3)	0 (0)		
Time undergoing ART, y					59.7	<.001
1.5–3	2650 (76.0)	513 (14.7)	319 (9.1)	5 (0.1)		
4–5	3712 (79.6)	525 (11.3)	420 (9.0)	9 (0.2)		
6–10	3685 (80.2)	499 (10.9)	402 (8.8)	7 (0.2)		
>10	1902 (82.6)	206 (8.9)	188 (8.2)	8 (0.3)		
Involvement in DSD					664.5	<.001
Yes	11 105 (81.4)	1354 (9.9)	1175 (8.6)	2 (0)		
No	844 (59.7)	389 (27.5)	154 (10.9)	27 (1.9)		

Data are presented as No. (%).

Abbreviations: ART, antiretroviral therapy; DSD, differentiated service delivery; NNRTI, nonnucleoside reverse transcriptase inhibitor; WHO, World Health Organization.

In multivariate analysis, predictors of having any viremia included not being enrolled in a DSD model (adjusted odds ratio [aOR], 2.60; 95% CI, 2.28–2.97), receiving ART for <3 years (aOR, 1.52; 95% CI, 1.32–1.73), and being 0 to 14 years of age (aOR, 1.21; 95% CI, .79–1.86; Table 4).

**Table 4.** Factors Associated With Viremia Among Clients With Initial Undetectable Viral Load in Southern Nigeria (N = 15 050)

	Unadjusted Odds Ratio (95% CI)	*P* Value	Adjusted Odds Ratio (95% CI)	*P* Value
Sex				
Male	1 [Reference]		…	…
Female	1.00 (.92–1.09)	.988		
Age category, y				
0–14	1 [Reference]		1 [Reference]	
15–24	0.65 (.52–.83)	<.001	0.70 (.55–.89)	.003
25–64	0.64 (.52–.79)	<.001	0.68 (.55–.85)	<.001
≥65	0.80 (.53–1.22)	.304	0.82 (.54–1.26)	.37
Education^a^				
None	1 [Reference]		…	…
Primary	0.99 (.83–1.17)	.881		
Postsecondary	1.12 (.95–1.30)	.173		
Marital status^a^				
Never married	1 [Reference]		…	…
Married	1.01 (.87–1.16)	.925		
Previously married	1.06 (.97–1.16)	.195		
Occupation^a^				
Employed	1 [Reference]		…	…
Unemployed	0.98 (.82–1.17)	.844		
Student	0.88 (.75–1.05)	.159		
WHO stage^a^				
I	1 [Reference]		…	…
II	1.04 (.94–1.15)	.442		
III	1.02 (.93–1.13)	.645		
IV	1.07 (.79–1.45)	.674		
Regimen				
Integrase inhibitors	1 [Reference]		…	…
Protease inhibitors	0.77 (.34–1.70)	.513		
NNRTIs	0.64 (.12–3.29)	.591		
Time undergoing ART, y				
1.5–3	1 [Reference]		1 [Reference]	
4–5	0.81 (.73–.90)	<.001	0.80 (.71–.90)	<.001
6–10	0.78 (.70–.87)	<.001	0.75 (.66–.85)	<.001
>10	0.66 (.58–.75)	<.001	0.61 (.52–.72)	<.001
Enrolled in DSD				
Yes	1 [Reference]		1 [Reference]	
No	2.83 (2.52–3.17)	<.001	2.60 (2.28–2.97)	<.001

Abbreviations: ART, antiretroviral therapy; DSD, differentiated service delivery; NNRTI, nonnucleoside reverse transcriptase inhibitor; WHO, World Health Organization.

^a^Complete data analyzed.

### Retention Outcomes

Of the 15 050 clients who had an undetectable initial VL, 98.6% remained active in treatment at the end of the follow-up period, while 1.4% interrupted treatment (0.7% were deceased, 0.6% were lost to follow-up, 0.1% stopped ART; Table 5).

**Table 5.** End-line Antiretroviral Therapy Status Among Clients With Initial Undetectable Viral Load in Southern Nigeria Stratified by Viral Load Categories

			Treatment Interruption			
	Total	Active	Stopped Treatment	Lost to Follow-up	Deceased	*P* Value
Viral load measure						.104
Durable viral suppression	11 949	11 797 (98.7)	10 (0.1)	72 (0.6)	70 (0.6)	
Transient viremia	1743	1743 (98.3)	8 (0.5)	8 (0.5)	14 (0.8)	
Persistent viremia	1329	1303 (98.0)	0 (0)	8 (0.6)	18 (1.4)	
Virologic failure	29	29 (100)	0 (0)	0 (0)	0 (0)	

Data are presented as No. (%).

## DISCUSSION

Our study aimed to evaluate the prevalence and factors associated with detectable viremia as well as the clinical and virologic outcomes of PWH undergoing ART who initially achieved viral suppression. In this study, the majority of clients (79.4%) maintained durable viral suppression, but 20.4% experienced viremic events during follow-up VL testing, with 0.9% ultimately developing virologic failure. Our results agree with findings from a study in Zimbabwe [23, 24], which used the same cutoffs (ie, undetectable levels classified as ≤50 copies/mL). These findings provide further evidence of the need for ongoing adherence support and regular monitoring even among people who achieve viral suppression after initiating ART. Ongoing adherence support and regular virologic monitoring will ensure timely identification of those with adherence challenges and will contribute to achieving durable virologic suppression for a higher number of those on treatment.

In contrast to studies reporting lower numbers of clients with durable viral suppression (34%–57%) [18, 25], nearly 80% of our clients maintained viral suppression. This may in part be explained by the high number of clients in our cohort who were enrolled into one of several DSD models [22, 26] that were widely implemented in this program. Clients who were not enrolled in a DSD model were 3 times more likely to be viremic than those who were in a DSD model.

Detectable viremia is associated with poor adherence [27]; other factors may result in detectable viremia. We did not have a reliable objective measure of adherence to ART in this study. Our study clearly indicates that the odds of clients being viremic decrease with longer duration of ART, highlighting the need for retention support in the earlier years following initiation.

Whereas Young et al reported higher virologic failure rates among PWH who experienced low-level viremia in a Swiss HIV cohort study [12], <1% of viremic cases progressed to virologic failure in our study cohort. The proportion of those developing virologic failure could be reduced with timely enhanced adherence counseling, a World Health Organization–recommended strategy that has been tested by other studies [28–30].

Our study had some limitations. First, we did not consider the effect of ART adherence on the incidence of viremia, owing to the lack of good objective measures of clients’ ART adherence. Also, we used routinely collected data, with the attendant risk of missingness, and only fewer variables collected. Despite these limitations, the large sample size strengthens our findings. To our knowledge, this is the first study in Nigeria to stratify VL by finer categories, which allowed study factors associated with any of these outcomes.

## CONCLUSION

In this study, the prevalence, treatment outcome, and risk factors associated with detectable viremia were investigated in a cohort of PWH undergoing ART in 2 southern states in Nigeria. Data from this study provide evidence for the need for ongoing adherence support even among clients who attain virologic suppression upon starting ART. According to our data, nearly 20% of PWH who initially attain virologic suppression subsequently developed a virologic event, and a few went on to develop virologic failure. Findings from this study point to priority subgroups for enhanced care, including those not enrolled in a DSD model and those with a shorter duration of ART.

## Notes

***Author contributions.*** U. U. A., S. I., and E. N. N. conceptualized the study. U. U. A., B. G., A. M. I., O. E. T., and T. B. conducted the data analysis with the advice of O. S., P. N., H. G. K., H. M. I., and M. H. B. All authors contributed to data interpretation, writing of the manuscript, and approval of the final version.

***Data availability statement.*** Data supporting the findings of this study are available from the corresponding author on request.

***Financial support*.** This work resulted in part from data collected during the implementation of the USAID-funded EpiC Project in Nigeria.
